# Proteomic Screening of Human Targets of Viral microRNAs Reveals Functions Associated with Immune Evasion and Angiogenesis

**DOI:** 10.1371/journal.ppat.1003584

**Published:** 2013-09-05

**Authors:** Amelia M. Gallaher, Sudipto Das, Zhen Xiao, Thorkell Andresson, Philippe Kieffer-Kwon, Christine Happel, Joseph Ziegelbauer

**Affiliations:** 1 HIV and AIDS Malignancy Branch, National Cancer Institute, National Institutes of Health, Bethesda, Maryland, United States of America; 2 Laboratory of Proteomics and Analytical Technologies, Advanced Technology Program, SAIC-Frederick Inc., National Cancer Institute at Frederick, Frederick, Maryland, United States of America; University of North Carolina at Chapel Hill, United States of America

## Abstract

Kaposi's sarcoma (KS) is caused by infection with Kaposi's sarcoma-associated herpesvirus (KSHV). The virus expresses unique microRNAs (miRNAs), but the targets and functions of these miRNAs are not completely understood. In order to identify human targets of viral miRNAs, we measured protein expression changes caused by multiple KSHV miRNAs using pulsed stable labeling with amino acids in cell culture (pSILAC) in primary endothelial cells. This led to the identification of multiple human genes that are repressed at the protein level, but not at the miRNA level. Further analysis also identified that KSHV miRNAs can modulate activity or expression of upstream regulatory factors, resulting in suppressed activation of a protein involved in leukocyte recruitment (ICAM1) following lysophosphatidic acid treatment, as well as up-regulation of a pro-angiogenic protein (HIF1α), and up-regulation of a protein involved in stimulating angiogenesis (HMOX1). This study aids in our understanding of miRNA mechanisms of repression and miRNA contributions to viral pathogenesis.

## Author Summary

Kaposi's sarcoma-associated herpesvirus is the virus associated with multiple proliferative disorders, including Kaposi's sarcoma, primary effusion lymphoma and multicentric Castleman's disease. This virus expresses small nucleic acids (with sequences distinct from other organisms), called microRNAs, that can limit expression of specific genes. Currently, we only know a few validated targets of these viral microRNAs and the mechanisms of microRNA-mediated repression are still being actively debated. We used a method to look at protein expression changes induced by these viral microRNAs to better understand microRNA targets and functions. The method we describe here found microRNA targets that are missed by other approaches. In addition to identifying previous microRNA targets and discovering new microRNA targets, we found the function of specific viral microRNAs to be associated with immune evasion and the expansion of blood vessel networks, a hallmark of Kaposi's sarcoma. The results may be a resource for those studying microRNAs from other organisms, and furthermore, the microRNA functions described provide mechanistic insight into viral pathogenesis and immune evasion.

## Introduction

At our current understanding, the herpesvirus family is the only viral family expressing multiple miRNAs. Kaposi's sarcoma-associated herpesvirus (human herpesvirus 8) expresses 12 pre-miRNAs [1], [2], [3], [4]. These miRNAs are encoded in the latency locus of the KSHV genome and all KSHV miRNAs are expressed during latency. This discovery presented the possibility that KSHV expresses miRNAs to modulate host gene expression by a mechanism that would avoid generating additional viral proteins, which could be detected by the host immune system.

Although many groups have been successful in detecting viral miRNA expression, our understanding of the functions of the viral miRNAs has been limited due to the small number of validated miRNA target genes. Previously identified human targets include thrombospondin [4], BACH-1 [5], [6], BCL-2 associated factor [7], MICB [8], musculoaponeurotic fibrosarcoma oncogene homolog [9], IκBα [10], Rbl2 [11], p21 [12], caspase 3 [13], TWEAKR [14], TGFβR2 [15], and other targets. These targets represent host genes involved in angiogenesis, transcription regulation, immune evasion, NF-κB regulation, epigenetic modifications, apoptosis and cell cycle regulation.

Recently, a number of other host targets have been identified by purifying RNA-induced silencing complexes and analyzing associated nucleic acids [16] [17], [18] in primary effusion cell lines, which represents a recent addition to the technologies used to identify miRNA targets. Gene expression studies to discover targets repressed by viral miRNAs in primary endothelial cells have been limited. Previous methods for miRNA target prediction include measuring changes at the mRNA level in response to miRNAs using microarrays and bioinformatic methods to search for limited sequence complementarity [4], [7]. The human targets of miRNAs that will be detected depend on the expression profiling methods utilized and the mechanisms of miRNA-mediated repression [19]. If a miRNA is inhibiting gene expression by stimulating deadenylation and destabilization of the mRNA target, then gene expression microarrays can be successful in identifying targets. However, miRNAs may repress gene expression of some targets by inhibiting translation and mRNA expression profiling may miss miRNA targets that are repressed at the protein level, but not at the mRNA level. One method to detect these types of targets is by measuring changes in protein expression in the presence of specific miRNAs. Stable isotope labeling of amino acids in cell culture (SILAC) coupled with tandem mass spectrometry has been used recently to study the effects of miRNAs on protein expression [20], [21], [22], [23]. In this report, the pulsed SILAC method was employed to focus on changes in newly translated proteins in the presence of KSHV miRNAs. Here, we report the discovery of human targets of viral miRNAs using this technology in primary human endothelial cells, a relevant cell type for KSHV infection. We found that specific miRNAs can inhibit expression of a protein involved in immune response and can stimulate expression of two proteins known to stimulate angiogenesis (a key hallmark of Kaposi's sarcoma).

## Results

**Figure 1:**
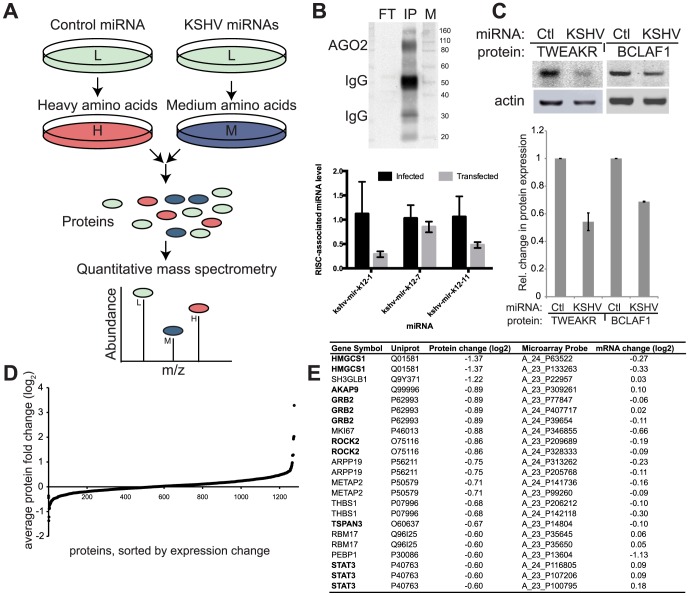
Proteomic screening for KSHV miRNA targets. (A) Experimental design shows HUVECs transfected with control or KSHV miRNAs, then labeled with stable isotope-labeled amino acids (normal/light “L”, medium-heavy “M”, and heavy “H”), cells from both conditions were combined and LC-MS/MS was used to measure relative abundance of peptides corresponding to the labeled amino acids. Green proteins symbolize proteins that were translated before the amino acid labeling and/or do not contain stable isotope-labeled amino acids. (B) Argonaute2 (AGO2) was immunoprecipitated from HUVECs. Western blot shows unbound lysate (flow-through, “FT”) and immunoprecipitated material (IP) probed with AGO2 antibody. Graph shows RT-PCR miRNA data from AGO2-immunoprecipiated material from at least three immunoprecipitations per sample from either KSHV-infected HUVECs (black) or HUVECs transfected with miRNA mimics (gray) as in pSILAC assay. (C). Known miRNA targets (TWEAKR and BCLAF1) are repressed when 16 miRNA mimics are co-transfected. Shown is two-color quantitative Western blot analysis from three biological replicates. (D) Range of relative changes in protein expression of all proteins detected with at least two peptides per protein and found in two biological replicates. (E) Table shows the most repressed proteins in the KSHV miRNA samples. Protein levels were determined by pulsed SILAC and mRNA levels were determined by microarray.

In order to identify the target genes repressed by KSHV miRNAs, we measured the effects of KSHV miRNAs on protein expression by introducing viral miRNAs into uninfected primary endothelial cells (HUVEC). Primary cells were transfected with either a control non-targeting miRNA mimic or a combination of sixteen KSHV miRNA mimics (Figure S1). We transfected miRNA mimics in the absence of viral infection to ensure that the repression of newly synthesized proteins was not an indirect result of infection or viral protein expression. After transfection, cells were grown in media supplemented with two distinct mixtures of stable medium-heavy or heavy amino acids. Using this pulsed labeling approach we measured newly translated proteins from the two conditions (Figure 1A) with mass spectrometry. The relative amount of mature miRNAs in RNA-induced silencing complexes was probed using Argonaute 2 immunoprecipitations, followed by reverse-transcription and quantitative PCR (normalized to human miR-21, which does not change upon KSHV infection [24]). Transfected miRNA mimics were indeed associated with the RNA-induced silencing complex (RISC) (Figure 1B) using this assay. Before mass spectrometry analysis, the samples were analyzed for expected repression of previously identified miRNA targets (BCLAF1, TWEAKR) using quantitative Western blotting (Figure 1C). Cells from the two labeling conditions were combined in a 1∶1 ratio, and proteins were extracted, fractionated and analyzed by tandem mass spectrometry to calculate relative changes in newly translated proteins due to the presence of KSHV miRNAs. Fractionation of the protein samples was utilized to improve coverage of a wide variety of proteins and to better detect less abundant proteins. Both biological replicates were analyzed by mass spectrometry twice, yielding two technical replicates for two biological replicates. Mass spectrometry data was filtered for proteins detected by at least two peptide pairs (medium-heavy and heavy) per replicate and detected in both biological replicates. There were 1276 proteins that met these stringent quality control filters (Figure 1D) and the most down-regulated proteins in cells containing KSHV miRNAs are shown in Figure 1E. It was noteworthy that thrombospondin (THBS1), the first identified target of KSHV miRNAs was strongly inhibited at the protein level [4].

### Enrichment of Seed-Matching Targets Corresponding to Repressed Proteins

**Figure 2:**
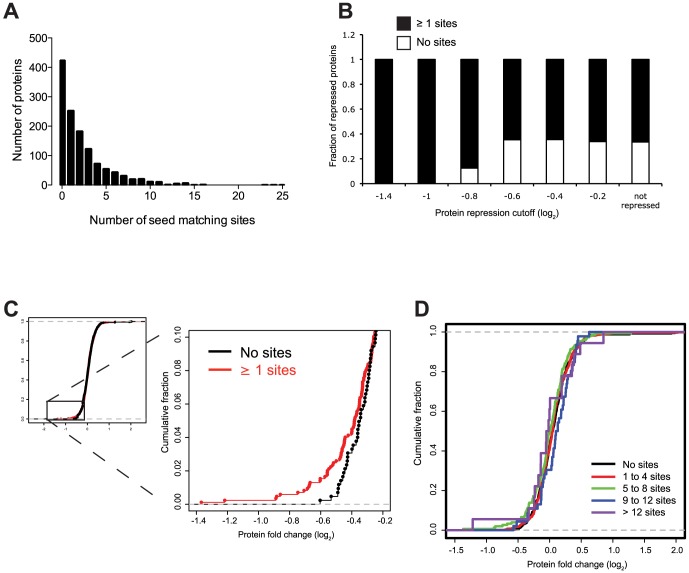
Analysis of miRNA seed-matching sites. (A) Proteins identified in screen were analyzed for KSHV miRNA seed-matching sites in their corresponding 3′UTRs using TargetScan. Histogram shows the distribution of the number of sites per 3′UTR. (B) Graph displays the fraction of proteins whose transcripts contain no or at least one seed-matching site in the transcripts of proteins with indicated repression levels in the presence of KSHV miRNAs. (C–D) Empirical cumulative distribution graph showing protein expression changes whose transcripts contain at least one miRNA seed-matching site (C) or classes of multiple sites (D).

Since sixteen miRNAs were introduced into HUVECs simultaneously during the SILAC assay, the analysis of potential miRNA targets and protein expression was complex, even though these experiments were biologically relevant to the expression of all miRNAs during normal viral infection. Bioinformatic programs are commonly used to identify complementary sequences between miRNAs and their potential targets. We used TargetScan [25] to search for seed-matching sequences in the 3′ untranslated regions (UTRs) of transcripts corresponding to proteins that were identified in the SILAC analysis. An initial analysis of genes included in both the SILAC and TargetScan datasets separated the genes into two sets, one with at least one TargetScan site (847 genes) and another set of corresponding transcripts which did not have any TargetScan sites (424 genes) (Figure 2A). This revealed that the fraction of proteins containing at least one predicted miRNA target site (in the corresponding transcript's 3′UTR) was larger in the set of proteins that were strongly repressed (Figure 2B). Approximately 60% of proteins that were not repressed (log_2_>0) had at least one seed-matching site in their corresponding 3′UTR, suggesting an over 60% false positive rate of detection using seed matching alone. However, those proteins whose transcripts have seed-matching sites tend to have lower expression in the presence of KSHV miRNAs, as do the proteins from mRNAs with multiple seed-matching sites (Figure 2C–D).

### Pulsed SILAC Identifies KSHV miRNA Targets

**Figure 3:**
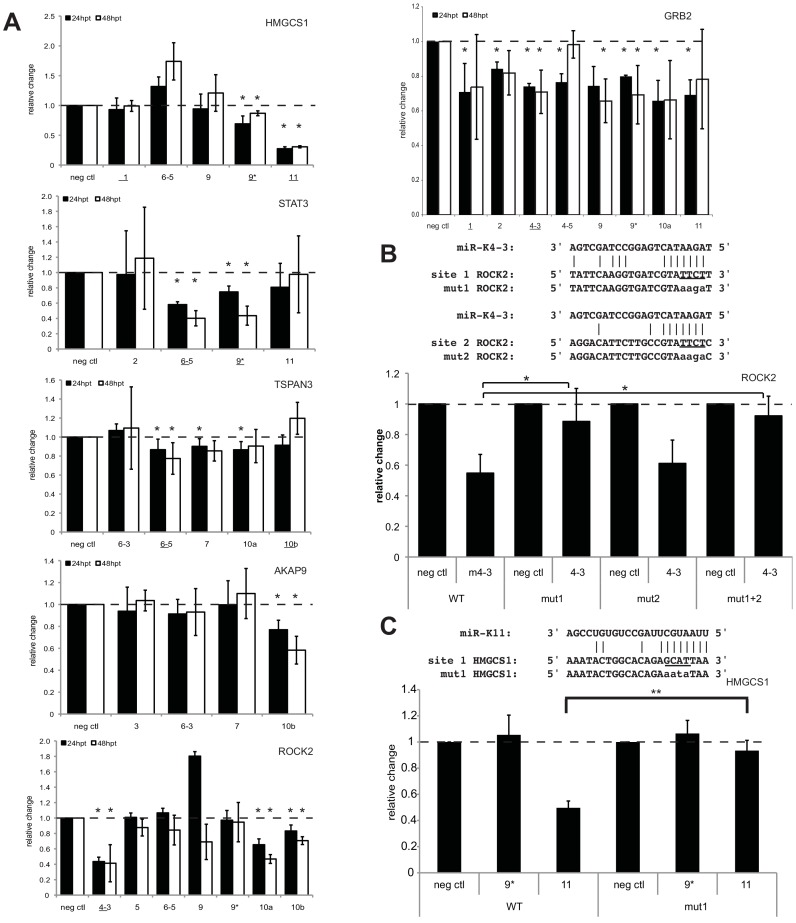
Validation of predicted miRNA target hits by 3′UTR luciferase assays. (A) Luciferase reporters with full-length 3′UTRs were co-transfected with miRNAs. Relative changes in luciferase activity were normalized to a negative control miRNA (neg ctl), an internal luciferase control, and a luciferase vector control without the cloned 3′UTR of interest. Underlined miRNAs were predicted targets based on seed matches in Table S2. (B–C) Predicted miRNA target sites were identified by TargetScan and mutated as shown. Luciferase results are shown for mutated ROCK2 (B) and HMGCS1 (C). Asterisks denote P<0.05 (*), or P<0.01 (**), n≥3 using a T-test.

Repressed proteins detected in the SILAC analysis can represent direct targets of KSHV miRNAs, as well as indirect targets. In order to determine if these repressed genes are directly targeted by KSHV miRNAs, we chose six genes based only on protein expression changes to test in standard 3′UTR luciferase reporter assays. Using full 3′ UTRs, we determined that all six of the 3′UTR luciferase reporters tested (GRB2, ROCK2, STAT3, HMGCS1, TSPAN3, AKAP9) are significantly inhibited by at least one KSHV miRNA (Figure 3A), but TSPAN3 repression was the weakest of the six 3′UTRs tested. Interestingly, GRB2 was also recently described as a target of KSHV miRNAs [17]. Additionally, we mapped the specific site targeted by a KSHV miRNA for two of these targets, ROCK2 and HMGCS1 (Figure 3B–C). Luciferase reporters shown in Figure 3A contained 3′UTRs downstream of a firefly luciferase gene and reporters shown in Figure 3B–C had 3′UTRs downstream of a renilla luciferase gene. Different transcription rates, half lives of luciferase enzymes, and cloned 3′UTR context may have been responsible for certain variations in the repression of the same 3′UTR in different reporter plasmids. The mutation of predicted sites significantly relieved miRNA-mediated repression for both miRNA targets (Figure 3B–C). Together, these results suggest the 3′UTRs of these six genes identified in the SILAC screen contain sequences targeted directly by KSHV miRNAs.

**Figure 4:**
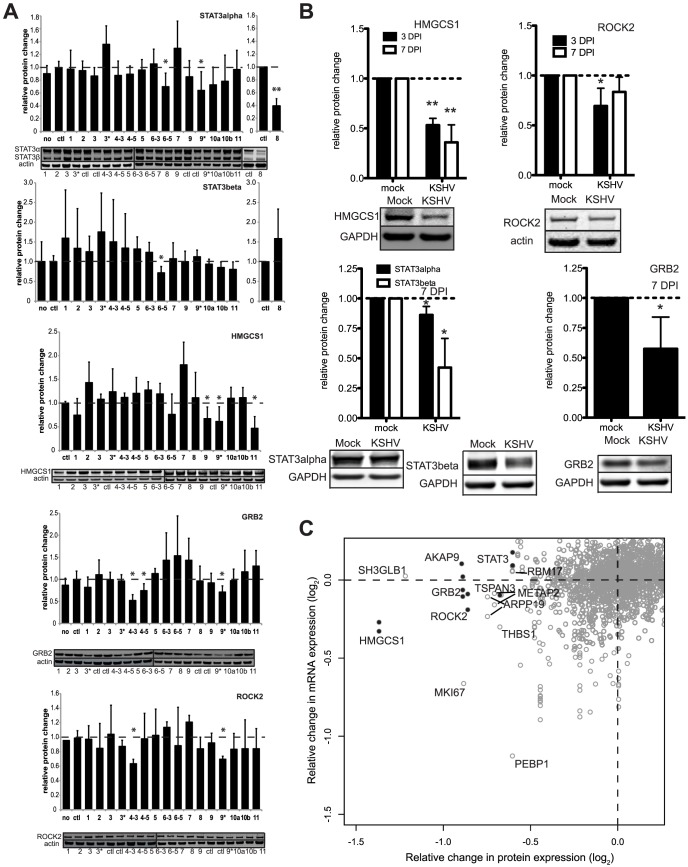
Validation of predicted miRNA targets with Western blotting and *de novo* infection. (A) Primary HUVECs were transfected without miRNAs (no), a negative control miRNA (ctl), or KSHV miRNAs. Whole cells lysates were analyzed using Western blotting and normalized to actin (loading control) and the negative control miRNA (ctl). MirVana miRNAs mimics for miR-K8 in STAT3 assays are shown. Other mirVana miRNA mimic results are shown in Figure S2. Average relative protein expression changes are shown with error bars showing S.D. from ≥3 biological replicates. (B) Primary HUVECs were *de novo* infected with KSHV (3 or 7 days post infection) and whole cell lysates were analyzed using Western blot analysis as in (A). Asterisks denote P<0.05, n≥3 using a T-test. (C) Plot showing average changes in protein expression on the horizontal axis from SILAC data and mRNA changes from microarray data (vertical axis) from the same transfections. Gray open circles are gene products found in both assays and black filled circles represent gene products from six validated targets (HMGCS1, STAT3, GRB2, ROCK2, AKAP9, TSPAN3). Multiple microarray probes are indicated for a subset of genes, yielding multiple vertically-aligned circles from multiple microarray probes, but one protein measurement (See Figure 1E for examples).

Using two-color quantitative Western blotting, we assayed sixteen mature miRNAs for their ability to modulate endogenous protein expression of four (of the six) luciferase-validated target genes in primary endothelial cells. All four proteins tested, GRB2, ROCK2, STAT3 (alpha and beta isoforms) and HMGCS1, were inhibited significantly by at least one miRNA (Figure 4A). Furthermore, the protein expression from the majority of the individual genes tested was inhibited significantly by multiple miRNAs. For example, GRB2 protein expression was repressed by miR-K4-3p, -K4-5p, and -K9*. We observed an overall correlation between the miRNAs that repress the 3′UTR reporter and the miRNAs that decrease the steady-state levels of endogenous protein. This supports the pulsed SILAC strategy as a method of discovering miRNA targets.

It is also important to determine target protein expression levels in the context of viral infection. We observed significant repression of four miRNA targets, including a particularly robust inhibition of HMGCS1 in *de novo* infected HUVECs compared with mock infected cells (Figure 4B). The repression of HMGCS1 protein after infection was similar to the protein expression changes in the pSILAC data (Figure 1E) and cells transfected with miR-K11 mimics (Figure 4A). Repression after *de novo* infection validates that these targets are repressed in the context of physiological levels of viral miRNAs during infection.

### Pulsed SILAC Identified Targets Missed Using Microarrays

An additional use of the proteomic data is to address the question of how miRNAs repress gene expression. Whether miRNA-induced gene expression changes are reflected primarily at the mRNA or the protein level may lead to a better understanding of miRNA repression mechanisms. Using the same transfected cells from the proteomic screening, we also analyzed the mRNA expression profiles using microarrays (Figure 4C, Table S1). All of the protein expression changes in Figure 1D were combined with mRNA expression changes from microarray analysis and plotted in Figure 4C. The protein and mRNA expression changes of the six newly validated miRNA targets were analyzed and for all six of these target genes the changes at the protein level were more pronounced than at the mRNA level (Figure 4C). These findings justified the additional focus on protein expression changes to predict miRNA targets, which may be missed by solely measuring changes at the mRNA level (depending on the mRNA expression change cutoff values used).

### Functional Significance of Select miRNA Targets

**Figure 5:**
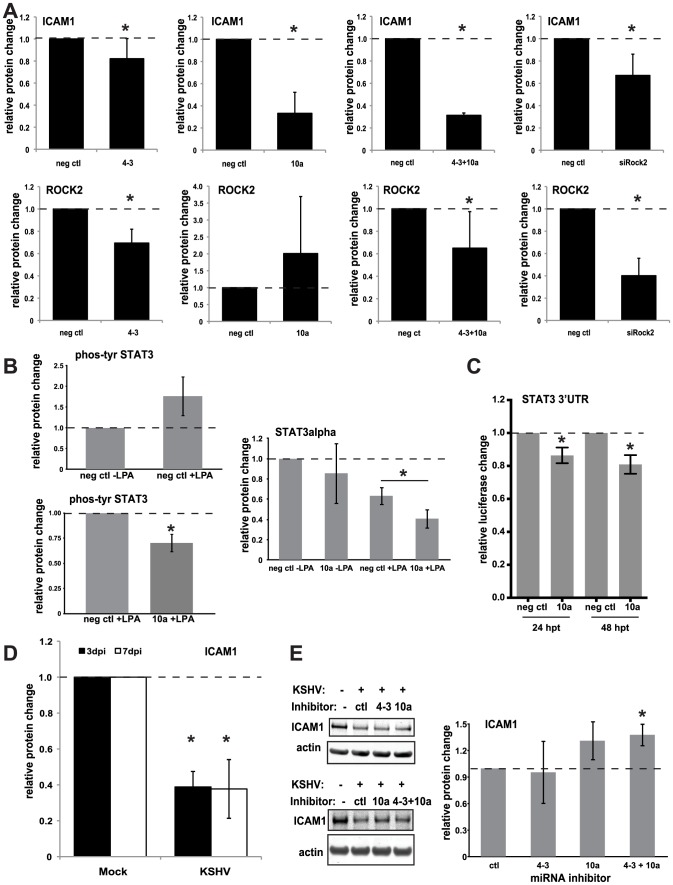
KSHV miRNAs repress *intercellular adhesion molecule 1* (ICAM1). (A) HUVECs transfected with control (neg ctl) or KSHV miRNAs were stimulated with lysophosphatidic acid (LPA) to activate ICAM1 protein expression. Shown are results from Western blot analysis of ICAM1 and ROCK2 protein levels (relative to internal control GAPDH) from LPA-treated cells. (B) Western blot analysis of the phosphorylation of Tyr705 of STAT3 and total levels of STAT3. (C) Luciferase assays with the STAT3 3′UTR were performed as in Figure 3. (D) HUVECs were infected with KSHV, and ICAM1 protein levels were measured by Western blot analysis. (E) HUVECs were transfected with miRNA inhibitors shown, infected with KSHV, and ICAM1 protein levels were determined by Western blot analysis. Average relative protein expression changes are shown with error bars showing S.D. from ≥3 biological replicates. Asterisks denote P<0.05 using a T-test.

Identifying potential miRNA targets is an initial step to elucidate the functions of KSHV miRNAs. One of the validated miRNA targets, Rho-associated, coiled-coil containing protein kinase 2 (ROCK2) has been shown to be largely responsible for lysophosphatidic acid (LPA)-induced intercellular adhesion molecule 1 (ICAM1) expression in HUVECs [26]. ICAM1 is essential for the recruitment and transmigration of leukocytes to sites of inflammation [27]. Therefore, we hypothesized that KSHV miRNA-mediated knockdown of ROCK2 would contribute to the decrease of ICAM1 expression induced by LPA as part of a host immune evasion strategy during latency. HUVECs were transfected with individual or combinations of KSHV miRNAs or siRNAs targeting ROCK2, treated with LPA, and harvested at 48 h post-transfection. The whole cell lysates were analyzed for relative changes in ROCK2 and ICAM1 protein expression by quantitative Western blot analysis. In LPA-treated cells, ROCK2 protein was sufficiently repressed by both miR-K4-3p and siROCK2, but not reproducibly by miR-K10a. We observed an average 6-fold increase of ICAM1 protein expression upon treatment with LPA (data not shown). While there was a significant decrease in ICAM1 protein expression from LPA-treated cells also transfected with miR-K4-3p or siROCK2, there was a much more robust repression of ICAM1 expression by miR-K10a transfection (Figure 5A). Based on these results, we hypothesized that miR-K10a represses ICAM1 up-regulation through a ROCK2-independent mechanism. It was known that STAT3 can activate ICAM1 expression [28], [29], [30] and LPA treatment induces STAT3 phosphorylation [31]. We confirmed an increase in phospho-STAT3 (Tyr705) using Western blot analysis upon LPA treatment and found decreased levels of phospho-STAT3 (Tyr705) in the presence of miR-K10a (Figure 5B). While repression of total STAT3 protein levels with miR-K10a transfection in the absence of LPA was variable, STAT3 protein levels were repressed in LPA-treated cells upon transfection with miR-K10a mimics compared to control mimics. TargetScan analysis found three potential miR-K10a binding sites in the STAT3 3′UTR (Table S2), and luciferase assays with the STAT3 3′UTR confirmed direct repression by miR-K10a (Figure 5C). This suggested a potential role of STAT3 in the repression of ICAM1 in LPA-treated endothelial cells that is independent of ROCK2. Additionally, we observed strong repression of ICAM1 after *de novo* KSHV infection in HUVECs (Figure 5D). To determine if KSHV miRNAs play a role in this repression, HUVECs were transfected with miRNA inhibitors to miR-K4-3p and miR-K10a, then infected with KSHV, and analyzed for ICAM1 protein expression three days after infection. ICAM1 protein expression is modestly elevated (likely due to incomplete inhibition of target miRNAs) in HUVECs transfected with miR-K4-3p and miR-K10a inhibitors (Figure 5E). Together, these results show that KSHV miRNAs decrease LPA-stimulated ICAM1 expression and are at least partially responsible for ICAM1 repression during KSHV infection in primary endothelial cells, which could potentially minimize recruitment of leukocytes to areas of KSHV infection.

Our initial focus was to identify direct miRNA target genes by focusing on genes that were repressed in the presence of the viral miRNAs. However, we were intrigued by the increased protein production of heme oxygenase 1 (HMOX1, log_2_ = 2.03) and biliverdin reductase (BLVRA, log_2_ = 1.99) in the presence of KSHV miRNAs. These proteins are important factors in oxidative stress and heme metabolism [32]. HMOX1 protein was previously described to be upregulated upon infection with KSHV [33]. Because miRNAs usually work through suppressing gene expression, these results suggested that some KSHV miRNAs may work through modulating protein expression of factors regulating HMOX1 and BLVRA protein expression.

**Figure 6:**
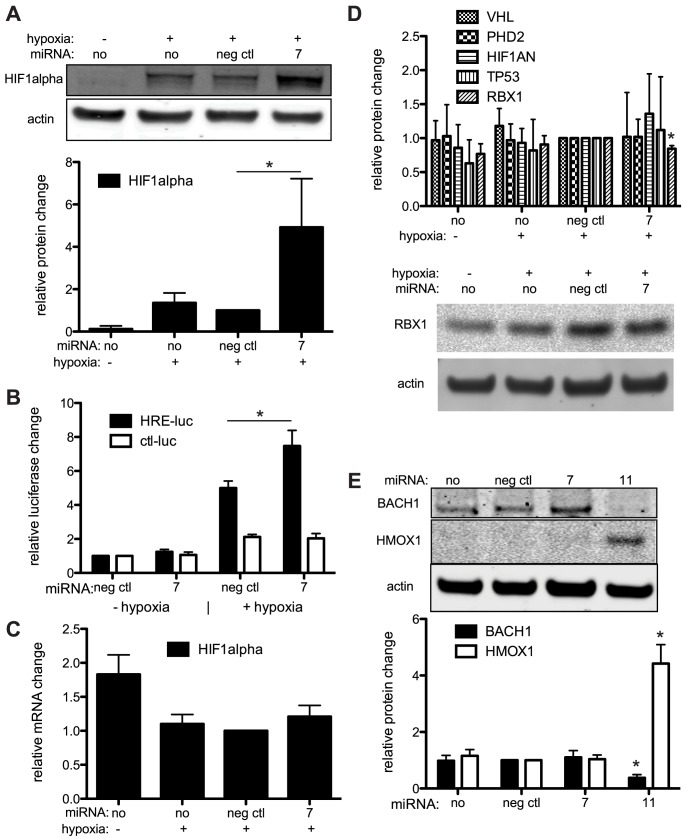
KSHV miRNAs increase HIF1α and HMOX1 protein levels. (A) Western blot analysis of cells transfected with miRNAs and exposed to hypoxia (blots above, quantitation below). (B) Luciferase reporters containing hypoxia responsive elements (HRE-luc) or parental control (ctl-luc) in the promoter were transfected into 293 cells with miRNAs and exposed to hypoxia (1% oxygen for 16 hr.). Luciferase activity was normalized to an internal control reporter as well as the condition without hypoxia and transfected with the negative control miRNA. (C) Cells were treated as in (A) and HIF1α mRNA was measured using qPCR. (D) The same samples used in (A) were analyzed by Western blot analysis for proteins shown. Data were analyzed and presented as in (A). (E) Cells were transfected with KSHV miRNAs or controls and analyzed for BACH1 and HMOX1 protein levels using Western blot analysis. Average relative protein expression changes are shown with error bars representing S.D. from ≥3 biological replicates. Asterisks denote P<0.05 using a T-test.

An analysis of promoters corresponding to the up-regulated proteins (top 5%) revealed that HIF1α binding sites were enriched in this set of up-regulated genes (p-value = 0.0005). Closer inspection revealed both HMOX1 and BLVRA are transcriptional targets of HIF1α [34], [35]. We sought to determine if specific miRNAs could influence HIF1α expression or activity. Inducing hypoxia in 293 cells (also HUVECs, data not shown) with the addition of a hypoxia mimic, cobalt chloride (data not shown), or inducing hypoxia with incubation in 1% oxygen, showed that miR-K7 can induce a 5-fold activation of endogenous HIF1α protein levels (Figure 6A). We also observed that miR-K7 can increase HIF1α transcriptional activity through assays using a HIF-responsive luciferase reporter (Figure 6B). Quantitative PCR data did not detect a significant change in HIF1α mRNA levels (Figure 6C), suggesting transcription rates are not affected by miR-K7. HIF1α protein is constitutively produced, but destroyed in cells growing in normoxic conditions. We suspected that miR-K7 might increase HIF1α protein levels by repressing an inhibitor of HIF1α protein expression. We investigated the changes in protein expression of four inhibitors of HIF1α, including hypoxia-inducible factor 1-alpha inhibitor (HIF1AN), egl nine homolog 1 (PHD2/EGLN1), von Hippel-Lindau tumor suppressor (VHL), and tumor protein p53 (TP53), but we did not detect significant changes (Figure 6D). However, another protein, ring-box 1/E3 ubiquitin protein ligase (RBX1), has been shown to mediate ubiquitination and degradation of HIF1α [36]. Protein levels of RBX1 were modestly repressed in hypoxic cells transfected with miR-K7 mimic compared to the negative control miRNA mimic (Figure 6D). It was unknown if RBX1 is a direct target of miR-K7, but RBX1 was found in miRNA target detection screens (CLIP assays) in KSHV-infected cells [17], [18]. These data suggested that RBX1 may play a partial role in miR-K7 upregulation of HIF1α protein levels during hypoxia, but it remains likely that up-regulation of HIF1α is due to changes in expression of multiple genes that remain to be determined. Taken together, these results suggest miR-K7 may repress additional inhibitors of HIF1α protein expression. In normoxia, HMOX1 protein expression was not induced by miR-K7 (Figure 6E). Furthermore, the increase in HMOX1 protein expression detected in the SILAC analysis (in normoxia) was likely not due to increased HIF1α protein levels, but rather repression of a repressor of HMOX1.

In addition to positive regulation by HIF1α, HMOX1 was also known to be repressed by BTB and CNC homology 1, basic leucine zipper transcription factor 1 (BACH1) which is a known target of miR-K11 [5], [6]. Under normoxia and miR-K11 expression, we observed an expected repression of BACH1 and a robust 4.5-fold activation of HMOX1 protein expression (Figure 6E). These results suggest up-regulation of HMOX1 by miR-K11 is achieved by repression of BACH1 during normoxia.

**Figure 7:**
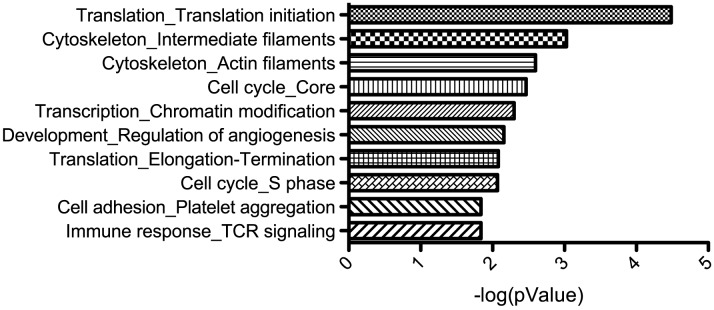
Enriched classes of proteins repressed by KSHV miRNAs. A list of the 5% most repressed proteins (Fig. 1C) was analyzed for the most enriched networks of interacting gene products using Metacore (GeneGo). This is a similar analysis to gene ontology term enrichment analysis.

In addition to determining the roles of miRNAs through the study of individual target genes, the analysis of predicted target gene functions could highlight cellular pathways and biological processes that miRNAs regulate during infection. Furthermore, repressed gene expression could be the result of direct or indirect consequences of miRNAs, but both classes of targets may influence KSHV-infected cells. Analysis of the biological processes enriched in the most repressed (five percent) proteins showed that many of these repressed proteins are involved in translation, cytoskeleton, cell cycle, chromatin modification and angiogenesis (Figure 7). While it is currently unknown how many of these repressed proteins are direct miRNA targets, this analysis points to certain cellular functions important to KSHV pathogenesis that KSHV miRNAs are targeting, directly or indirectly.

## Discussion

In order to understand miRNA functions, it is critical to identify their targets, so we can increase our knowledge of cellular pathways that are important for infection and pathogenesis. Genome-wide studies have been conducted analyzing the Argonaute-associated mRNAs (CLIP assays) in B cells [16], [17], [18], and the microarray and proteomic screening for miRNA-induced gene expression changes in primary endothelial cells from this report represent a complimentary dataset for elucidating viral miRNA functions. Indeed, integration of miRNA targets from CLIP methods and other expression studies will continue to be useful for identifying miRNA target sites, as well as >those CLIP hits that are repressed at the mRNA and/or protein level. Compared with other approaches to discover miRNA targets, current mass spectrometry methods are able to query a lower number of gene products. Despite this limitation, this current study has identified repression of multiple novel and previously validated miRNA targets (THBS1, GRB2). Additionally, gene expression studies can reveal direct and indirect miRNA targets, both of which are important for virus-host interactions. By inspecting gene expression changes at both the mRNA and protein level, we have demonstrated that multiple miRNA targets are likely missed using microarrays since the miRNA target may only be repressed at the level of translation. This finding is relevant given the conflicting reports about the predominant mechanism and order of repression mechanisms [37] that are utilized by miRNAs to modulate gene expression, whether that be mRNA level repression [38] or translation inhibition [39], [40]. In this study, validated miRNA targets AKAP9, STAT3, and GRB2 proteins were significantly repressed, but microarray results indicated mRNA levels were not reduced in the presence of KSHV miRNA mimics. The protein SH3-domain GRB2-like endophilin B1 (SH3GLB1) was the second most inhibited protein, but the mRNA levels were relatively unchanged (log_2_ 0.03). Interestingly, previous reports have shown that SH3GLB1 functions as a tumor suppressor and pro-apoptotic factor [41], [42]. Given our findings, this proteomic method is clearly an important start to discover novel miRNA targets. Furthermore, we have also shown novel functions of viral miRNAs involved in cellular pathways important to KSHV pathogenesis, including ICAM1 repression, HMOX1 up-regulation and HIF1α up-regulation.

Previous studies have indicated that ROCK2 is involved in a pro-inflammatory pathway induced by lysophosphatidic acid (LPA) that results in the up-regulation of intercellular adhesion molecule 1 (ICAM1) on the surface of endothelial cells [26]. ICAM1 binds with lymphocyte function-associated antigen 1 (LFA-1) and leads to the recruitment and transmigration of leukocytes. Interestingly, ICAM1 is downregulated from the cell surface and degraded through a well-described mechanism by the KSHV lytic protein, K5, which can cause a decrease in the recruitment of helper T cells [27], [43], [44]. Furthermore, a previous study [45] and this report have also shown a decrease in ICAM1 expression during latent de novo infection of endothelial cells. We discovered that KSHV miRNAs, miR-K10a and miR-K4-3p, repress ICAM1 expression after induction by LPA, likely through ROCK2 and STAT3-associated pathways. Our data indicate that miR-K10a may be inhibiting LPA induction of ICAM1 by multiple mechanisms. First, the repression of a direct or indirect miRNA target of miR-K10a may be partially responsible for the decrease in LPA-induced STAT3 phosphorylation. HITS-CLIP data [18] showed the kinase PTK2B/FAK as a hit for miR-K10a alone, and, interestingly, PTK2B/FAK is thought to be responsible for phosphorylation of STAT3 in LPA-treated cells [31]. Although STAT3 protein levels can be repressed by miR-K6-5p, unlike miR-K10a, it is not predicted to target the kinase (PTK2B/FAK) and it remains to be determined if miR-K6-5p can repress LPA-activation of ICAM1. Second, miR-K10a may directly inhibit STAT3α total protein levels in LPA-treated cells, as suggested by the results from the STAT3 3′UTR luciferase assays with miR-K10a. While others [45] have shown that low levels of the KSHV protein K5 can still down-regulate ICAM1 expression, we believe it is likely that during latent infection, the inhibition of ICAM1 is also due to the viral miRNAs, miR-K4-3p and miR-K10a. However, further studies are required to further elucidate the contributions of viral protein and viral miRNA-mediated repression of ICAM1.

HIF1α can activate transcription of VEGF and other factors involved in angiogenesis [46], which raises the possibility that KSHV miRNAs may influence the angiogenic environment in KSHV-infected endothelial cells. Since miR-K7 increases HIF1α protein levels, but did not inhibit some major repressors of HIF1α (Figure 6), this suggests miR-K7 is working through an alternative pathway. We also observed a modest decrease in RBX1 when HIF1α is upregulated and the combined data suggest that there may be an underappreciated mechanism regulating HIF1α protein levels. Others have reported an increase in HIF1α activity with KSHV infection [47], [48], [49]. This increased activity is likely due to contributions from both viral proteins and viral miRNAs.

Interestingly, analysis using MetaCore software reveals human genes involved in translation initiation are enriched in the proteins repressed by KSHV miRNAs in endothelial cells. This class of translation initiation genes was also enriched in predicted miRNA targets from both KSHV and EBV miRNAs in co-infected latent BC1 cells [17], [50]. By contrast, lytic viral infections have been known to repress host translation inhibition [51] and others report that translation is activated upon KSHV lytic reactivation [52]. Together, these results suggest KSHV may play a complex role in influencing translation during latency and lytic infection. This investigation into HIF1α regulation by miRNAs was raised by the fact that the HIF1α transcriptional target heme oxygenase (HMOX1) is strongly upregulated in this proteomic screen and in KSHV infected cells in a previous report [33]. It was also found that increased HMOX1 activity stimulated proliferation of KSHV-infected endothelial cells [33]. Both heme oxygenase I (HMOX1) and bilverdin reductase (BLVRA) are strongly up-regulated in the presence of KSHV miRNAs in our study, and both of these gene products can protect endothelial cells from oxidative stress [53]. This also suggests certain KSHV miRNAs may protect cells from oxidative stress, by inhibiting BACH1 from repressing HMOX1 expression. Increased HMOX1 activity also correlates with increased angiogenesis [54], [55], [56], [57]. Taken together, KSHV miRNA induction of HMOX1 can potentially protect cells from oxidative stress and increase proliferation and angiogenesis. In summary, the SILAC method revealed miRNA targets and discovered ways in which KSHV miRNAs can influence proliferation, angiogenesis, and immune evasion. More in-depth studies are needed to fully understand the significance of selected human genes targeted for repression by viral miRNAs.

## Materials and Methods

### Cell Culture and Reagents

293 cells were maintained in Dulbecco's modified Eagle's medium (DMEM) containing 10% fetal bovine serum (FBS) and 1× penicillin and streptomycin (Pen Strep) glutamine solution (Gibco). Primary human umbilical vein endothelial cells (HUVECs; Lonza) were maintained in EGM-2 (Lonza) for up to five passages. Locked nucleic acids were from Exiqon. Synthetic KSHV miRNA mimics and a non-targeting miRNA (control) were from Ambion (Sequences in Supplemental Information). HUVECs were seeded at 2×10^5^ cells/well in a 6-well plate, transfected by using 1.5 µl/well DharmaFECT 1 reagent (Dharmacon) and 10 nM KSHV miRNA, and harvested at 48 h posttransfection (hpt). ON-TARGETplus SMARTpool small interfering RNAs (siRNAs) targeting ROCK2 and an ON-TARGETplus nontargeting pool were obtained from Dharmacon. For ICAM1 experiments, cells were then serum starved overnight in basal media (EBM-2) with 25% EGM-2 and, 40 hours post-transfection, treated with LPA (50 µM, Enzo) for 8 hours. Cells were harvested at 48 hr. post-transfection and lysed in RIPA. For SILAC experiments, HUVECs were transfected (total miRNA concentration was 10 nM) in T75 flasks for 6 hr. and then split into new flask with medium-heavy (with ^13^C_6_-L-arginine and D_4_-L-lysine) or heavy (^13^C_6_^15^N_4_ L-arginine and ^13^C_6_^15^N_2_ L-lysine) SILAC media as described [20], except the media was also supplemented with endothelial growth factors (Bulletkit, Lonza). After 30 hr. post-transfection, cells were harvested from flasks, counted, and equal number of cells from each condition were combined and frozen.

### Trypsin Digestion and Peptide Fractionation

Frozen cell pellet containing equal amount of control (neg miRNA) and experimental (KSHV miRNAs) cells were suspended in 100 µl of 25 mM ammonium bicarbonate buffer (pH 8.4). The cells were lysed by brief sonication and the proteins were denatured by heating the protein lysate at 95°C for 5 min. Protein concentration was estimated using standard BCA assay (Pierce) and the lysate was subjected to trypsin (enzyme to protein ratio 1∶100) digestion overnight at 37°C. The tryptic digest was lyophilized and reconstituted in 25% ACN/0.1% FA (100 µl) and fractionated using strong cation exchange (SCX) liquid chromatography into 96 fractions. The fractions were pooled on the basis of the intensity profile into 45 fractions, vacuum dried and reconstituted in 12 µL of 0.1% formic acid prior to nano-flow reversed-phase liquid chromatography mass spectrometry analysis.

### Nanoflow Reversed Phase Liquid Chromatography Tandem Mass Spectrometry (nanoRPLC-MS/MS)

NanoRPLC–MS/MS analysis was performed using an Agilent 1100 nanoflow LC system coupled with hybrid linear ion trap-fourier transform ion cyclotron resonance (LIT-FTICR) mass spectrometer (LTQ FT Ultra) (ThermoElectron, San Jose, CA). The system was connected to a 75 µm i.d.×360 mm o.d.×10 cm long fused silica microcapillary column (Polymicro Technologies, Phoenix, AZ) packed in-house with 5 µm, 300 Å pore size C-18 silica-bonded stationary RP particles (Vydac, Hysperia, CA). The LC mobile phase A was 0.1% formic acid in water and B was 0.1% formic acid in acetonitrile. After the peptide sample injection, gradient elution was performed under the following conditions: 2% B at 500 nL/min in 30 min; a linear increase of 2–42% B at 250 nL/min in 40 min; 42–98% B at 250 nL/min in 10 min; and 98% at 500 nL/min for 18 min. The LIT-FTICR-MS was operated in the profile mode with 50,000 resolution for FTMS scans and followed by the data-dependent MS/MS scans where the seven most abundant peptide molecular ions in each FTMS scan were sequentially selected for collision-induced dissociation (CID) using a normalized collision energy of 35%. Dynamic exclusion was applied to minimize repeated selection of peptides previously selected for CID. The capillary temperature and electrospray voltage were set to 160°C and 1.7 kV, respectively.

### SILAC Data Analysis

The raw LC-MS/MS data obtained from FT-LTQ was analyzed by MaxQuant (version 1.0.13.13) for peptide identification and quantification. MS/MS peak list from individual RAW files were generated using the Quant module of the MaxQuant software and protein identification was performed using MASCOT against a decoy human database. Oxidation of methionine was searched as a variable modification. The false discovery rate was set at 1% for peptide and protein identification. Peptide peak intensities were used to determine the relative abundance ratio of “heavy” labeled proteins to “medium” labeled proteins. Unlabeled peptides were not used for further analysis. The ratio of “heavy” to “medium” proteins represents the fold change values reported (Figure 1D-E, Tables S1, S2). Raw data files from pSILAC from both technical replicates were combined and then processed in MaxQuant to improve the coverage and the number of peptides found per protein. The Spearman correlation coefficient between protein expression changes for the two biological replicates is 0.51 (and 0.43 Pearson correlation coefficient). The Spearman correlation coefficient between mRNA expression changes for the two biological replicates is 0.54 (and 0.57 Pearson correlation coefficient). Due to the limited amount of sample obtained from the primary cells, equal amount of heavy (H) and medium (M) labeled cells were mixed prior to processing of the samples. To verify that there was no labeling bias, an MA plot (M = log2(H)−log2(M), A = ½(log2(H)+log2(M)) was performed followed by Lowess curve analysis on the transformed data. Figure S4 shows that the Lowess regression line is almost straight around zero horizontal line, demonstrating no labeling bias in the H and M labeling.

### Microarrays

RNA was purified using Tri reagent (Ambion) and RNA quality was determined using a Bioanalyzer 2100 (Agilent). Agilent arrays were performed and analyzed using Agilent Feature Extraction Software and Genespring GX as previously [7]. HUVEC microarray data was deposited to NCBI GEO database, accession number GSE43640.

### Western Blot Analysis

Total cell protein was harvested from cell pellets by using RIPA lysis buffer (Sigma) supplemented with 1× Halt protease and phosphatase inhibitor cocktail (Thermo Scientific). Cells were lysed on ice for 10 min, and cell debris was removed by centrifugation at 13,000 rpm for 10 min. Nuclear extracts for HIF1α blots were prepared using NE-PER (Pierce). The Li-Cor Odyssey system was used for the detection and quantitation of protein bands. The following primary antibodies were used: rabbit anti-TWEAKR (4403, Cell Signaling), rabbit anti-BCLAF1 (Bethyl), goat anti-BACH1 (SC-14700, Santa Cruz), mouse anti-GAPDH (sigma), anti-STAT3 (9132S, Cell Signaling), rabbit anti-HMGCS1 (sc-33829, Santa Cruz), rabbit anti-GRB2 (3972S, Cell Signaling), rabbit anti-ICAM1 (4915, Cell Signaling), rabbit anti-ROCK2 (sc-5561, Santa Cruz), mouse anti-HIF1α (NB100-105, Novus) and mouse anti-actin (AC-74, catalog number A5316; Sigma) antibodies. The following secondary antibodies conjugated to infrared (IR) fluorescing dyes were obtained from Li-Cor: goat anti-rabbit antibody IR800CW, goat anti-mouse antibody IR680, and goat anti-mouse antibody IR800CW. Protein band intensities were calculated and background corrected using ImageStudio (Li-Cor). Results are normalized to actin levels, relative to levels in mock-infected or negative-control miRNA conditions.

### Luciferase Assays

Full-length 3′UTR assays were performed as previously described [58]. Assays in Figure 3A used 3′UTR firefly luciferase reporters and were contransfected with a control renilla luciferase reporter under the control of a thymidine kinase promoter. Assays in Figure 3B–C used 3′UTRs cloned into a dual luciferase reporter. The 3′UTRs were cloned downstream of the renilla luciferase gene reporter. Luciferase values were normalized to an internal luciferase reporter and to parental vectors lacking cloned 3′UTRs. The hypoxia-inducible factor (HIF) luciferase reporter has five HIF-responsive elements in the promoter upstream of firefly luciferase reporter gene (Panomics). Mutations of the predicted miRNA binding sites within the 3′UTRs of ROCK2 and HMGCS1 were performed as previously described [58] using the following primers and their reverse compliments: 5′-GCAGGCCTGCAAATACTGGCACAGAAATATAATCATACACCTTATTAACGGTGA-3′ for HMGCS1 and 5′-CTATGAAAGCAGTCATTATTCAAGGTGATCGTAAAGATCCAGTGAAAACAAGACTGAAATAT-3 for ROCK2 mut1 and 5′-TTACGCAGGACATTCTTGCCGTAAAGACATGATCCCAGATAAGTGTGTGT-3′ for ROCK2 mut2.

### Argonaute 2 (Ago2) Immunoprecipitation and miRNA Detection

HUVECs were transfected as in SILAC experiments (mixture of 16 mimics, total concentration of mimics was 10 nM). Each Ago2 immunoprecipitation was performed from individual T75 flasks using an Ago2 antibody (20 µl per 1 ml immunoprecipitation of diluted lysate, Cell Signaling #2897), and the Magna RIP System (Millipore). Purified RNA was subjected to TaqMan MicroRNA Reverse Transcription Kit. Mature miRNA levels were determined using Taqman MicroRNA Assays and viral miRNAs were normalized to human miR-21 levels using the ΔΔCt method. Note uninfected and untransfected HUVECs (control) had no detectable miR-K12-1, but displayed average threshold cycles of 35 for miR-K12-7 and 37 cycles for miR-K12-11.

### Sequence Analysis

Uniprot IDs from pSILAC data, Agilent microarray probe IDs, and TargetScan v5.0 Refseq IDs were mapped to Ensembl gene IDs. Data integration was performed using Ensembl gene IDs. TargetScan sites “8mer”, “7mer-m8”, and “7mer-1A” were included, but 6mer sites were not included. Additional seed matching information is provided in Table S2. Data similar to Table S2 was used to calculate the number of seed matching sites per 3′UTR. The empirical cumulative distribution function (ecdf) was performed using R (http://CRAN.R-project.org/). Promoter analysis of hypoxia-inducible factor responsive elements was performed using ExPlain (Biobase) using up-regulated (top 5%) proteins compared to a control set of genes normally expressed in HUVECs.

### Statistical Analysis

At least three biological replicates were used for each analysis and the mean and standard deviation were used in T-tests. Changes were considered statistically significant when P<0.05.

### Accession Numbers

ENSG00000134318

ENSG00000168610

ENSG00000140391

ENSG00000127914

ENSG00000112972

ENSG00000177885

## Supporting Information

Figure S1 miRNA mimic sequences used in this study.
(DOC)

Figure S2 Luciferase and Western blotting with mirVana microRNA mimics. (A–B) Luciferase reporters with full-length 3′UTRs were co-transfected with miRNAs. Relative changes in luciferase activity were normalized to a negative control miRNA (neg ctl), an internal luciferase control, and a luciferase vector control without the cloned 3′UTR of interest. Asterisks denoted P<0.05 (*), P<0.01 (**), n≥3 using a T-test. (C) Primary HUVECs were transfected without miRNAs (no), with a negative control miRNA (ctl), or with KSHV miRNA mimics. Whole cells lysates were analyzed using Western blotting and normalized to actin (loading control) and the negative control miRNA (ctl). Average relative protein expression changes are shown with error bars showing S.D. from ≥3 biological replicates. Numbers shown next to protein names reflect the weight (kilodalton) of each protein.
(EPS)

Figure S3 Plots denote protein and mRNA expression changes shown in Figure 4C, but gene products are separated into two plots depending on if TargetScan detected a seed match site in the corresponding 3′UTR (“with seeds” or “without seeds”).
(EPS)

Figure S4 (A) MA Plots for SILAC biological replicates (M = log2(H)−log2(M), A = ½(log2(H)+log2(M)) was performed followed by lowess curve analysis on the transformed data. The lowess regression line (in red) is almost straight around 0, demonstrating no labeling bias in the Heavy isotope and Medium isotope labeling. (B) Histogram showing data from Figure 1D with Gaussian fitted curve in red. Inset graph shows a magnified section of repressed proteins.
(EPS)

Figure S5 Mass spectrometry spectra for three proteins (HMGCS1, AKAP9, GRB2) mentioned in Figure 1. Each spectra shows the relative abundance for the light, medium-heavy, and heavy version of a specific peptide from each protein.
(EPS)

Table S1 Average fold changes (log2) from two biological replicates for pSILAC or microarrays. The fold change values reflect the difference from the KSHV miRNA samples versus miRNA negative control samples. Gene products are identified with Uniprot, Agilent microarray probe number, and official gene symbol. RNA fold change was determined by microarray and protein fold change was determined by pSILAC (nanoRPLC–MS/MS).
(XLS)

Table S2 RNA and protein expression changes with seed matching data. The microarray and pSILAC data from Table S1 was integrated with output from TargetScan v5.0 (http://www.targetscan.org/vert_50/). The transcript sequence bases with predicted targets of KSHV miRNAs are shown with references to the multiple sequence alignment (MSA_start) and the human 3′UTR (UTR_start) sequences. The sequence files are available on the TargetScan website.
(XLS)
