# Supplementary material for: Proteomic Screening of Human Targets of Viral microRNAs Reveals Functions Associated with Immune Evasion and Angiogenesis
Source: PLoS Pathog. 2013 Sep 5;9(9):e1003584. doi: 10.1371/journal.ppat.1003584 (PMC3764211; doi:10.1371/journal.ppat.1003584)
Supplement: Figure S1 — miRNA mimic sequences used in this study. (DOC) [file ppat.1003584.s001.doc]

Figure S1

KSHV miRNAs from Ambion used in pSILAC screen using sequences in miRBase at the time of the screen. The mature miRNA-12-8 sequence was later updated in miRBase and the updated version was used in the Western blots for STAT3 in Figure 4.

>12-1

AUUACAGGAAACUGGGUGUAAGC

>12-10a

UAGUGUUGUCCCCCCGAGUGGC

>12-10b

UGGUGUUGUCCCCCCGAGUGGC

>12-11

UUAAUGCUUAGCCUGUGUCCGA

>12-2

AACUGUAGUCCGGGUCGAUCUG

>12-3

UCACAUUCUGAGGACGGCAGCG

>12-3*

UCGCGGUCACAGAAUGUGACA

>12-4-3p

UAGAAUACUGAGGCCUAGCUGA

>12-4-5p

AGCUAAACCGCAGUACUCUAGG

>12-5

UAGGAUGCCUGGAACUUGCCGG

>12-6-5p

CCAGCAGCACCUAAUCCAUCGG

>12-6-3p

UGAUGGUUUUCGGGCUGUUGAG

>12-7

UGAUCCCAUGUUGCUGGCGCU

>12-8

UAGGCGCGACUGAGAGAGCACG

>12-9*

ACCCAGCUGCGUAAACCCCGCU

>12-9

CUGGGUAUACGCAGCUGCGUAA

KSHV miRNA used in Figure 4A (Ambion mirVana)

>12-8

CUAGGCGCGACUGAGAGAGCA
